# Uterine Rupture at 21 Weeks in Twin Pregnancy with TTTS and Previous C-Section

**DOI:** 10.1155/2017/2690675

**Published:** 2017-08-03

**Authors:** Gieta Bhikha-kori, Marieke Sueters, Johanna M. Middeldorp

**Affiliations:** Fetal Therapy, Department of Obstetrics, Leiden University Medical Center, Leiden, Netherlands

## Abstract

Uterine rupture is a health problem in every country. The diagnosis is not always obvious and fetal and maternal morbidity and mortality can be high.

## 1. Introduction

Uterine rupture is defined as the complete disruption of all uterine layers, including the serosa. Undiagnosed and untreated, this is a life-threatening complication for mother and fetus. Other adverse outcomes related to uterine rupture are bladder laceration, severe hemorrhage, hysterectomy, and neonatal morbidity related to intrauterine hypoxia.

A large majority of uterine rupture occurs in the setting of a vaginal birth after caesarean (VBAC), resulting in intrapartum injury and spontaneous rupture of a gravid uterus. Out of hospital trauma represents the remainder of etiologies for uterine rupture. Motor vehicle accidents, domestic violence, and falls are the most common causes of blunt trauma during pregnancy.

The incidence of C-section in 2010 was 25.2% in North Europe and 32% in the United States. The C-section rate in the Netherlands increased from 7.4% in 1990 to 16.3% in 2012 [1, 2].

The incidence of uterine rupture in women with a previous C-section is 0.2–1.5% [3–9]. The incidence in the Netherlands was 0.64% in the period of 2004 till 2006 [10].

Several factors are known to increase the risk of uterine rupture. Contractions in spontaneous labor and the induction and augmentation of labor are the most important. Other known factors are maternal age, advanced gestational age, birth weight > 4000 gram, and interpregnancy interval < 18–24 months [9–12]. Twin pregnancy should also be considered a risk factor [13, 14].

Here we report a case of uterine rupture at 21 weeks of gestation in a patient with a previous C-section and currently pregnant with a monochorionic twin pregnancy complicated by twin to twin transfusion syndrome (TTTS) and premature contractions.

Besides TTTS polyhydramnion can be a risk factor for developing an uterine rupture.

## 2. Case

A 33-year-old woman was referred to our tertiary center (Leiden University Medical Center, the national referral center for fetal therapy in the Netherlands), because of a monochorionic pregnancy complicated by TTTS. In her obstetrical history she had a HELLP syndrome and intrauterine growth restriction and a C-section at 31 + 2 weeks of gestation.

In the current pregnancy she had prenatal screening with no associated high risk and an advanced structural echo with no abnormalities. For further summarization of the pregnancy, see Table 1.

At gestation of 21 + 5 the patient became anxious and hypotensive, with a blood pressure of 60/30 mm Hg and a pulse of 105 beats per minute.

At examination the uterus was tense on palpation with no signs of contractions. Ultrasound examination revealed two vital fetuses with a normal adjacent lying placenta. Again, some intraperitoneal free fluid was seen. At vaginal examination no vaginal blood loss was seen and the cervix was 1 cm dilated. Also a fluid challenge of 2 liter ringers solution and natrium chloride was given. The hemoglobin count was 7.8 (9.1) g/dl, prothrombin time slightly prolonged (16.1 seconds), and fibrinogen was normal (3.3 g/L). The differential diagnosis was threatening labor or uterine rupture or abruption.

An epidural for pain relief was given and the dilatation progressed to 2 cm.

Hemodynamically the patient continued to be compromised (RR 100/60 and pulse 120); despite a fluid challenge (now in total 4,5 L ringers and natrium chloride solution alternately) the Glasgow coma scale (GCS) was 15/15. Furthermore she developed fever (38,1 Celsius), for which antibiotics were administered.

The differential diagnosis was broad: uterine rupture, infected hematoma, or intrauterine infection. A CT abdomen was planned. Before transportation to the radiology department the patient became acutely instable with loss of consciousness, a very low blood pressure of 60/30, and a high pulse rate of 150 beats per minute. Laparotomy was planned immediately.

**Figure 1:**
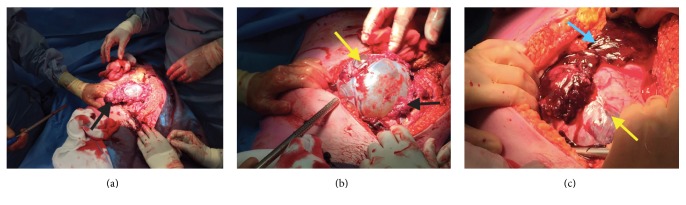
Laparotomy: after midline incision the uterus rupture on the right side with the bulging amniotic sac was visible (yellow arrows), the placenta partially protruding on the edges of the rupture wound (black arrows) with blood clots in the free abdominal cavity (blue arrow).

Explorative laparotomy was conducted through a midline incision. A uterine rupture at the right side of the uterine scar was diagnosed with necrotic/fibrotic wound edges, the placenta was partially protruding on the edges, and two dead fetuses were born (Figures 1(a), 1(b), and 1(c)). The insertion of the placenta was not in the scar. There were no signs of placenta percreta.

The rupture was closed in 2 layers with a total blood loss of 4000 ml. Transfusion of 10 packed cells and 2 units of fresh frozen plasma was given. During the procedure platelet count was 131, the prothrombin time (PT) was 17.0 seconds (slightly prolonged) and the fibrinogen was 1.8 g/L (low).

After operation the patient recovered gradually. She was discharged seven days after laparotomy. Hemoglobin was 8.37 g/dl at dismission.

## 3. Discussion

In monochronic twin pregnancies there is a 10–15% chance of developing TTTS.

Very little has been published on uterine ruptures in TTTS pregnancies, as this is the third case since 2004. In 2004 Tutscheck [15] reported a uterine rupture in a twin pregnancy with TTTS at a gestational age of 19 weeks. This case report describes a uterine rupture in a patient with a C-section through a corporal incision in the obstetric history [15].

The second case report from Smid in 2015 describes a 42-year-old woman, with a C-section in her obstetric history, with a posterior uterine rupture at 21 weeks of gestation with stage 1 TTTS [16].

Also dichorionic twin pregnancies can be complicated by uterine rupture.

Greenwald et al. described a case of asymptomatic uterine dehiscence in the second trimester of a dichorionic twin pregnancy diagnosed at 19 weeks with ultrasound [17]. The other case report was from Lana Saciragic who described a postpartum diagnosed uterine rupture after vaginal delivery of a dichorionic twin at 32 weeks of an unscarred uterus [18].

In our case the hypothesis is that at the gestational age of 16 weeks there already was a small dehiscence, explaining the free fluid seen at ultrasound investigation. Also at 12 weeks there is a great suspicion that the dehiscence was present if we look at the free fluid at ultrasound investigation and complaint of pain in the lower abdomen. This dehiscence grew while the gestation progressed, and the premature contractions in combination with the polyhydramnion due to TTTS augmented this process into a uterine rupture.

The first signs were already present at 12 weeks. The first change in the maternal condition was the hypotension, progressing to shock in hours ahead.

The free fluid beside the uterus with a normal condition of fetuses did not raise the suspicion of a uterine rupture at first, although it was in our differential diagnosis. The reassuring condition of the fetuses especially did not plead for uterine rupture, and the focus was on threatening labour or an abruption placentae. Fever raised the suspicion of an infection. This created the delay in the diagnosis because of this peculiar presentation of this case. The acute hypotension with loss of consciousness was a clear indication of an acute incident very suspicious for an uterine rupture, followed by a laparotomy.

Multiple signs/symptoms can precede a uterine rupture; these are abdominal pain (69%), fetal heart rate abnormalities (67%) [9, 10], vaginal bleeding (27%), hypertonia of the uterus (20%), and abrupt cessation of contractions (14%) [10]. In most cases the combination of abdominal pain and fetal heart rate abnormalities is present [9–11].

In our case there was hypertonia of the uterus with 2 vital fetuses and signs of contractions and vaginal blood loss.

The risk factors for uterine rupture in our case were a C-section in the obstetric history at preterm gestation of 31 weeks, a twin pregnancy, and TTTS with polyhydramnion. With preterm C-section there would be probably a corporal incision because a lower uterine segment has not been developed. The necrotic/fibrotic wound edges indicate a chronic process, as we did not see signs of an infection. An explanation is that the scar defect was present for a longer time but not bleeding actively and that this scar defect got bigger as a result of TTTS and polyhydramnion.

What we have to learn from this case is that TTTS with polyhydramnion (with or without contractions) can be a risk factor for uterine rupture. We know that 80.5% of uterine ruptures occur in women with a trial of labour [10]. The presence of contractions (and the loss of it acutely) is not always necessary to consider uterine rupture as a diagnosis, as a “silent” rupture can occur in women with TTTS and polyhydramnion without contractions. In fact, in every case of spacious uterine distention we have to keep a uterine rupture in mind.

## 4. Conclusion

Uterine rupture is one of the rare complications of TTTS that can appear in a twin pregnancy, especially when other risk factors such as a uterine scar, with or without contractions, are present. Also polyhydramnion in the setting of the TTTS should be considered a risk factor. Furthermore, as we know of earlier reported cases [17], we also have to consider uterine dehiscence in women with a uterine scar and TTTS.

## Figures and Tables

**Table 1:** 

Gestational age (weeks)	Symptoms	Physical/plan
12	Pain in her lower abdomen	Ultrasound examination: some free fluid was seen beside the uterus and a small hematoma in the fundus of the uterus. The explanation for the free fluid was thought to be a corpus luteum bleeding.

16	Trauma, fall on right side of her abdomen	In ultrasound examination some free fluid was seen intraperitoneally. Hemoglobin level was 8.85 g/dl.

21 + 2	Presentation with vaginal bleeding	The ultrasound was normal and no signs of TTTS were seen; the cervical length was 15–17 mm.

21 + 3	There was an increase in contractions and pain	A threatening TTTS Quintero stage 1 was diagnosed: fetus 1 showed a deepest vertical pocket (DVP) of amniotic fluid of 79 mm and fetus 2 a DVP 19 mm. Stomach and bladder filling were present in both fetuses and Dopplers were normal. The cervical length was 15 mm. There was not yet an indication for fetoscopic laser coagulation of the vascular anastomosis. Tocolysis with indomethacin was started.

21 + 4	There was minimum of painless brown vaginal bleeding	Ultrasound examination showed signs of TTTS Quintero stage 1, with a DVP of 10 cm in fetus 1 and a DVP of 1.9 cm in fetus 2. Stomach and bladder filling were normal in both fetuses as were the Dopplers. There was an anterior localization of the placenta. Because of TTTS Quintero 1 with cervical shortening a laser procedure was planned for the next day.

21 + 5		See further.
